# Estimation of Pneumonic Plague Transmission in Madagascar, August–November 2017

**DOI:** 10.1371/currents.outbreaks.1d0c9c5c01de69dfbfff4316d772954f

**Published:** 2018-11-01

**Authors:** Maimuna S. Majumder, Emily L. Cohn, Mauricio Santillana, John S. Brownstein

**Affiliations:** Computational Epidemiology Group, Division of Emergency Medicine, Boston Children’s Hospital, Boston, MA, United States; Institute for Data, Systems, and Society (IDSS), Massachusetts Institute of Technology, Cambridge, MA, United States; Computational Epidemiology Group, Division of Emergency Medicine, Boston Children’s Hospital, Boston, MA, United States; Department of Pediatrics, Harvard Medical School, Boston, MA, United States; Department of Pediatrics, Harvard Medical School, Boston, MA, United States; Computational Epidemiology Group, Division of Emergency Medicine, Boston Children’s Hospital, Boston, MA, United States; Department of Pediatrics, Harvard Medical School, Boston Children’s Hospital, Boston, MA, United States

**Keywords:** Madagascar, plague

## Abstract

Introduction: Between August and November 2017, Madagascar reported nearly 2500 cases of plague; the vast majority of these cases were pneumonic, resulting in early exponential growth due to person-to-person transmission. Though plague is endemic in Madagascar, cases are usually bubonic and thus result in considerably smaller annual caseloads than those observed from August–November 2017.

Methods: In this study, we consider the transmission dynamics of pneumonic plague in Madagascar during this time period, as well as the role of control strategies that were deployed to curb the outbreak and their effectiveness.

Results: When using data from the beginning of the outbreak through late November 2017, our estimates for the basic reproduction number range from 1.6 to 3.6, with a mean of 2.4. We also find two distinctive periods of “control”, which coincide with critical on-the-ground interventions, including contact tracing and delivery of antibiotics, among others.

Discussion: Given these results, we conclude that existing interventions remain effective against plague in Madagascar, despite the atypical size and spread of this particular outbreak.

## Discussion

Between August and November 2017, Madagascar reported nearly 2500 suspected, probable, and confirmed cases of plague 1. Though plague is endemic in the island country, the annual total is typically about 400 – primarily bubonic – cases 2. Only 15% of cases associated with the August–November 2017 outbreak were bubonic, and an unusually large fraction of pneumonic (pulmonary) plague, which can be spread from person-person, resulted in early exponential growth 1. Given the atypical size and spread of the outbreak, concerns regarding the adequacy of existing interventions for the remainder of the plague season have arisen 3. Here, we describe the transmission dynamics of pneumonic plague in Madagascar from August to November 2017; we then consider the role of control strategies that were deployed to curb the outbreak and discuss their effectiveness.

To assess transmission dynamics over time, the Incidence Decay and Exponential Adjustment (IDEA) model was used to estimate the basic reproduction number (*R*_0_), discount parameter (*d*), and observed reproduction number (*R*_Obs_) at various serial intervals (*t*) 4^,^^5^^,^^6^. Given the limited size and scope of previous plague outbreaks in Madagascar (2), the affected population was assumed to be fully susceptible in August 2017. A serial interval length (*l*) of 3–7 days and an epidemic curve of pneumonic plague cases from the World Health Organization (WHO) were used to parameterize the model (Figure 1A) 7^,^^8^. Because initial emergence of pneumonic plague is considered rare 9, person-to-person transmission was assumed exclusively.

Estimates for *R*_0_ and *d* were computed using nonlinear optimization to minimize the sum of squared differences between the WHO-reported and modeled cumulative epidemic curves (*I*):

**Figure:** Modeling the cumulative epidemic curve, *I*. Here, *R*_0_ is the optimized basic reproduction number, *d* is the optimized discount parameter, and *t* is the serial interval through which the curve is estimated.

\begin{equation*}I=\sum_{t=0}^{t}{\left[ \frac{R_{0}}{(1+d)^t}\right]^t} \end{equation*}

*R*_0_ and *d* were then used to calculate the observed reproduction number for any given *t*:

**Figure:** Modeling the observed reproduction number, *R*_Obs_. Here, *R*_0_ is the optimized basic reproduction number, *d* is the optimized discount parameter, and *t* is the serial interval through which is *R*_Obs_ estimated.

\begin{equation*}R_{Obs}=\frac{R_0}{(1+d)^t} \end{equation*}

While *R*_0_ represents potential transmissibility of a given pathogen in a fully susceptible population, *R*_Obs_ represents observed transmissibility as population-level susceptibility changes throughout an outbreak 4^,^^5^. In this sense, *R*_Obs_ is much like the effective reproductive number (*R*_E_), which represents transmissibility in populations that are not fully susceptible 5.

**Panels A–B:**
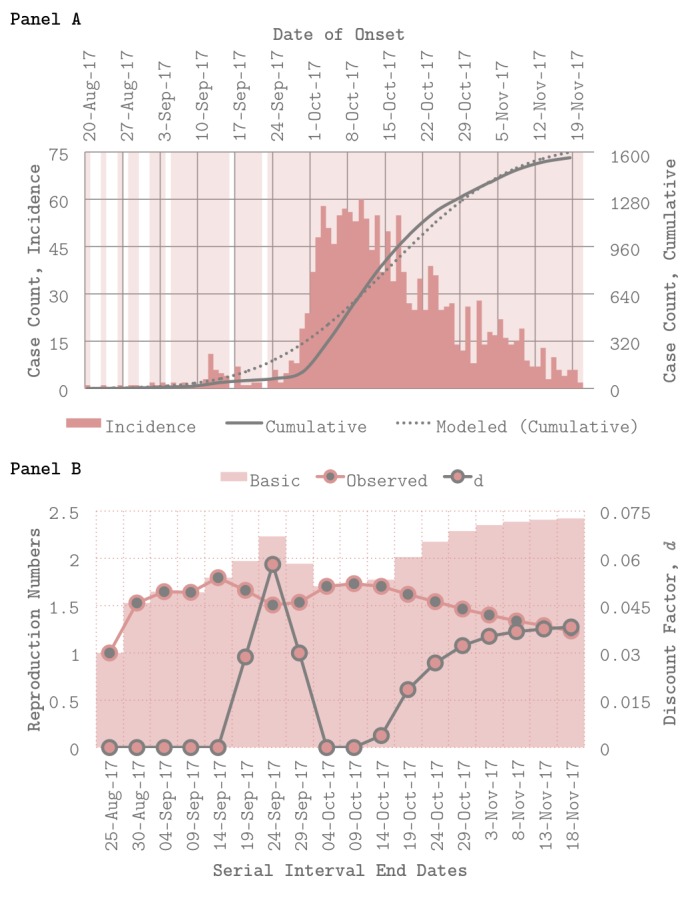
Epidemic curve, model fits, and transmission parameter estimates. Panel A (top) shows incidence and cumulative case counts of pneumonic plague over time by date of onset via the World Health Organization (WHO), as well as model fits for *R*_0_ (basic reproduction number) = 2.4, estimated using a serial interval length (l) of 5 days. Panel B (bottom) depicts *R*_0_ (basic reproduction number), *R*_Obs_ (observed reproduction number), and d (discount factor) at various serial intervals t, demonstrating changes in transmission dynamics over time.

When using data from the beginning of the outbreak through November 2017, *R*_0_ ranges from 1.6 to 3.6 with a mean of 2.4 (*l* = 5 days), which is consistent with other estimates for pneumonic plague 8^,^^10^. Model fits for *R*_0_ = 2.4 are shown in Figure 1A. When *R*_0_, *d*, and *R*_Obs_ are estimated using data from* t* = 0 (August 20, 2017) to *t* = 1, 2, … 18, time-sensitive trends emerge (Figure 1B). Figure 1B shows changes in all three parameters over time (*l* = 5 days). Here, larger values of the discount parameter (*d*) capture sharper decreases in observed transmissibility (*R*_Obs_), not only due to the natural depletion of susceptible individuals over time but also due, very likely, to behavior change and interventions put in place (i.e. control) during the course of the outbreak 5.

Two notable periods of control identified by the model – where *d* > 0 – are immediately apparent from Figure 1B: the first (Period 1), between September 14th and October 4th and the second (Period 2), from October 9th to November 18th. Period 1 is characterized by a linear increase – then decrease – in *d*, while Period 2 is characterized by a gradual, sub-linear increase in *d*. Interestingly, Period 1 coincides with two quickly-timed events: initial field investigations by the Madagascar Ministry of Public Health (MOPH), which were launched following identification of the index case on September 11th; and initial deployment of WHO staff to support on-the-ground response on September 18th 1. Meanwhile, Period 2 follows a carefully-planned series of events that took place from October 6th to October 9th, during which Médecins Sans Frontières deployed staff to support on-the-ground response (including social mobilization and community engagement efforts), the World Health Organization delivered 1.2 million doses of antibiotics to the Madagascar MOPH, and community healthcare workers were trained to conduct contact-tracing 1.

Because this particular outbreak was dominated by pneumonic plague transmission, spread primarily occurred via respiratory droplets 1. This, paired with the population density of the coastal cities that were most effected, likely explain the high rates of transmission (i.e. basic reproduction number nearing *R*_0_ = 3) estimated throughout the outbreak 7. Nevertheless, it appears that the aforementioned containment efforts – which focused not only on contact-tracing and antibiotic deployment but on preventative behavior change via risk communication and awareness campaigns as well – corresponded with rapid decreases in transmission of pneumonic plague during the August–November 2017 outbreak (Figure 1A). This success may be attributed at least in part to existing system memory in Madagascar’s healthcare infrastructure from previous – albeit smaller – plague outbreaks in the past 1^,^^2^. However, given that plague is endemic in the affected regions and occurs seasonally per annum even in the exception of outbreaks such as that studied here, continued investment in surveillance is key 1^,^^9^.

## Corresponding Author

Maimuna S. Majumder: maimuna@mit.edu

Phone: 1-978-460-3677

Mailing Address: 77 Massachusetts Avenue, Cambridge, MA, 02142

## Data Availability

All of the data used in this paper can be found directly in the references and are fully open source. The epidemic curve (which was used to parameterize the Incidence Decay and Exponential Adjustment [IDEA] model) was digitized from 7 and data regarding the interventions that were deployed can be found in 1.

## Competing Interests

The authors declare no competing interests.
